# Monoclonal Antibodies in Cancer Therapy

**DOI:** 10.3390/antib9030034

**Published:** 2020-07-20

**Authors:** David Zahavi, Louis Weiner

**Affiliations:** 1 Tumor Biology Training Program, Lombardi Comprehensive Cancer Center, Georgetown University, Medical Center, 3800 Reservoir Rd NW, Washington, DC 20007, USA; djz8@georgetown.edu; 2 Department of Oncology, Lombardi Comprehensive Cancer Center, Georgetown University, Medical Center, 3800 Reservoir Rd NW, Washington, DC 20007, USA

**Keywords:** monoclonal antibody, cancer, immunology, antibody-dependent cellular cytotoxicity (ADCC), immune checkpoint blockade (ICB)

## Abstract

Monoclonal antibody-based immunotherapy is now considered to be a main component of cancer therapy, alongside surgery, radiation, and chemotherapy. Monoclonal antibodies possess a diverse set of clinically relevant mechanisms of action. In addition, antibodies can directly target tumor cells while simultaneously promoting the induction of long-lasting anti-tumor immune responses. The multifaceted properties of antibodies as a therapeutic platform have led to the development of new cancer treatment strategies that will have major impacts on cancer care. This review focuses on the known mechanisms of action, current clinical applications for the treatment of cancer, and mechanisms of resistance of monoclonal antibody therapy. We further discuss how monoclonal antibody-based strategies have moved towards enhancing anti-tumor immune responses by targeting immune cells instead of tumor antigens as well as some of the current combination therapies.

## 1. Introduction

Antibodies were first described as a neutralizing substance found in blood by Behring and Shibasaburo in 1890 in their work on animal models of diphtheria [1]. Over the next century several important scientific advances would pave the way for the use of antibodies as a cancer therapeutic. Antibodies were identified as proteins that could recognize specific antigens by Heidelberger and Avery, and in 1947 Astrid Fagraeus demonstrated that antibodies were produced by plasma B cells of the adaptive immune system [2,3]. Sir Gustav Nossal then proved the clonal selection theory that a single B cell clone produces one specific antibody [4]. Thus, monoclonal antibodies (mAbs) are antibodies made by clones of a unique B cell, all of which bind to specific portions of an antigen also known as an epitope. Methods to produce monoclonal antibodies involving human–mouse hybrid cells were first identified by Schwaber in 1973 and were used by Köhler and Milstein to generate the human-derived hybridomas that have since been a mainstay in the large scale production of therapeutic antibodies [5,6]. Soon after the discovery of hybridomas, research into the use of mAbs to treat cancer began. Anti-melanoma mAbs were shown to suppress the growth of human melanomas in nude mice and in 1980 the first human trial of mAb therapy against cancer was conducted in a lymphoma patient [7,8]. Unfortunately, due to the murine origins of early therapeutic monoclonal antibodies, these mAbs were immunogenic in humans and were poor inducers of immunity in patients, thereby limiting their clinical applicability. In the late 1980s, techniques emerged to humanize antibodies in order to eliminate these limitations [9]. Further advances have led to the derivation of “fully-human” antibodies using transgenic mice or in vitro yeast or phage display systems [10,11]. As a result of these antibody engineering innovations, mAbs have become an important modality in the treatment of cancer. Antibodies are unique in their ability to both directly kill tumor cells while simultaneously engage the host immune system to develop long-lasting effector responses against the tumor. The combination of a multifaceted mechanism of action with target specificity distinguishes mAb therapy from treatments such as chemotherapy and underlies the capability of antibodies to elicit strong anti-tumor responses while minimizing toxicity and adverse events. To better understand how monoclonal antibodies function as a cancer therapy, we review their structure, mechanisms of action, clinical uses, mechanisms of resistance, and some combination strategies.

## 2. Antibody Structure and Function

Antibodies are large glycoproteins that belong to the immunoglobulin (Ig) superfamily and their role in the immune system is to recognize foreign antigens, neutralize them, and elicit a further immune response. Their basic structure is composed of two heavy and two light chains in the shape of a Y. At each tip of the Y lies the fragment antigen-binding (Fab) portion of the antibody which is responsible for recognition of the specific antigen. The fragment crystallizable (Fc) region located at the base of the Y structure mediates interactions between the antibody and other members of the immune system [12]. Antibody Fc regions are recognized by Fc receptors (FcRs) found on a wide range of immune cells. Based on the type of heavy chain, antibodies can be separated into five distinct classes: IgA, IgD, IgE, IgG, and IgM. IgG is the most often form used in antibody therapy due to the fact that IgGs interact with their associated type of FcR, FcγR, found on natural killer (NK) cells as well as neutrophils, monocytes, dendritic cells, and eosinophils to mediate specialized functions such as antibody-dependent cellular cytotoxicity (ADCC) and complement-dependent cytotoxicity (CDC). The IgG class can further be subdivided based on the ability of the Fc region to facilitate those functions: IgG1 and IgG3 are able to elicit ADCC and CDC, while IgG2 and IgG4 cannot [13]. Monoclonal antibodies represent a clonal version of a specified antibody isotype that is targeted to a unique antigen epitope.

## 3. Effector Mechanisms of Targeted mAbs

**Figure 1:**
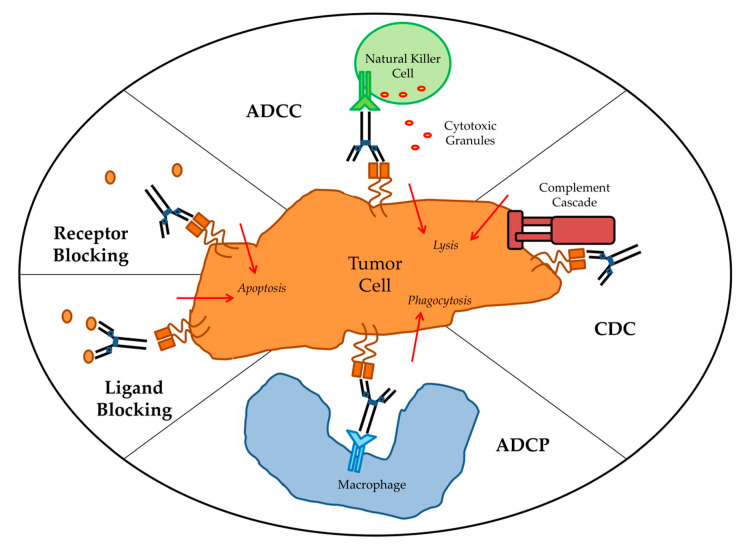
Antibody effector mechanisms. ADCC: antibody-dependent cellular cytotoxicity; CDC: complement-dependent cytotoxicity; ADCP: antibody-dependent cellular phagocytosis.

Targeted mAbs against antigens either unique to or overexpressed by tumor cells can cause tumor cell death by a variety mechanisms (Figure 1). The main direct mechanism by which many antibodies induce tumor cell death is the blockade of growth factor receptor signaling. Pro-tumor growth and survival signaling is perturbed when mAbs bind their target growth factor receptors and manipulate their activation state or block ligand binding. For example, epidermal growth factor receptor (EGFR) is overexpressed by many different cancers and signaling via EGFR leads to tumor cell proliferation, migration, and invasion. Cetuximab, which is an anti-EGFR mAb, induces apoptosis in tumor cells by blocking ligand binding and receptor dimerization [14,15]. Human epidermal growth factor receptor 2 (HER2) is a tyrosine kinase receptor that is overexpressed in many cancers but primarily ovarian and breast carcinomas [16]. It is distinct from EGFR in that it has no known ligand and instead hetero-dimerizes with other growth factor receptors to enhance their activation [17]. Antibodies targeting HER2 therefore achieve signaling perturbation by inhibiting hetero-dimerization and internalization. Trastuzumab was the first FDA approved anti-HER2 mAb and remains a vital component of treatments for *HER2*-amplified breast cancer. Indirect mechanisms of action of mAbs require the engagement of components of the host immune system and are CDC, antibody-dependent cellular phagocytosis (ADCP), and ADCC. Most targeted mAbs are able to activate the complement system. For instance, rituximab depends in part on CDC for its in vivo efficacy. In a preclinical model, rituximab anti-tumor effects were completely abolished by knockout of the complement cascade component C1q [18]. The importance of CDC in mAb therapy is further supported by the fact that genetic polymorphisms in the C1qA gene correlate with clinical response to rituximab in patients with follicular lymphoma [19]. Likewise, optimization of CDC via antibody engineering can enhance anti-tumor activity. For example, the anti-CD20 mAb ofatumumab, which mediates amplified CDC, demonstrated greater efficacy than rituximab in a clinical trial of chronic lymphocytic leukemia (CLL) patients [20]. ADCP occurs when FcγRI expressed on cells such as macrophages binds to IgG1 or IgG3 mAbs that have opsonized a tumor cell. There have been very limited studies of ADCP; however, there is some evidence that ADCP plays an important role in destruction of circulating tumor cells following mAb therapy [21]. First described in 1965 by Erna Möeller, ADCC has since been established as an immune mechanism where target cells become opsonized by antibodies which then recruits effector cells to induce target cell death by non-phagocytic mechanisms [22]. Antibodies act as bridges between by binding to antigens on the target cell surface via their Fab portions and linking the effector cells via their Fc portions. While IgG, IgA, and IgE can all mediate ADCC, IgG1 is the most relevant subclass for anti-cancer therapeutic antibodies [23]. Effector cells must express FcR that will bind the antibody in order to facilitate ADCC [24]. Each class of antibody has a corresponding class of FcR such as FcγR, which binds IgG, and FcαR, which binds IgA. FcγR is the most relevant class to ADCC of tumor cells and encompasses both the activating FcγRI (CD64), FcγRIIA (CD32A), FcγRIIIA (CD16A), and inhibitory FcγRIIB (CD32B) receptors [25]. When an activating FcγR on an effector cell binds the Fc region of an antibody receptor crosslinking and downstream signal propagation occurs. NK cells are the main effector type that mediate ADCC; however other myeloid types such as monocytes, macrophages, neutrophils, eosinophils, and dendritic cells are also capable [26]. Effector cells induce target cell death via cytotoxic granule release, Fas signaling, and initiation of reactive oxygen species [27,28,29]. While several myeloid cell types have been demonstrated to mediate ADCC during immunotherapy, the clinical efficacy of most targeted mAbs is mainly NK cell dependent [30].

While many mAbs facilitate several of the above mechanisms there has been debate about which mechanisms are important in vivo. Many of the first mAb therapies were known to mediate ADCC of tumor cells in vitro, but whether ADCC was significant to their therapeutic efficacy was originally poorly understood. Using mouse models, Clynes et al. were the first to demonstrate that ADCC was an essential contributor to the in vivo activity of trastuzumab and rituximab [31]. Additional mechanistic studies utilizing similar mouse models confirmed that FcγR expression by immune effector cells is required for tumors to respond to mAb therapy [32]. Furthermore, in a novel mouse model whose immune cells had mutant FcγR incapable of ADCC, mAb therapy failed to clear tumors [33]. In most of these studies the mAbs used could also act through additional mechanisms of action such as signaling perturbation. Therefore, while these results indicated ADCC was required for successful mAb therapy they did not prove whether ADCC alone was sufficient. More recent work involving mAbs that rely exclusively on ADCC have verified that ADCC alone can mediate therapeutic benefit [34]. In humans, clinical trials have demonstrated that many mAbs eliminate tumor cells, in part, by causing ADCC. Because functioning FcγRs were critical for mAb efficacy in mouse models, clinical trial data was used to examine whether FcγR polymorphisms would correlate with clinical outcomes. In humans, bothFcγRIIA and FcγRIIIA are polymorphic, with certain genotypes coding for FcγRs with higher affinity for IgG1 and therefore stronger ADCC activity [35,36]. Lymphoma patients who had the polymorphism associated with augmented ADCC were demonstrated to have better clinical responses to rituximab in several studies [37,38,39]. Furthermore, both genotypes of FcγRIIA and FcγRIIIA associated with higher affinity for Fc were robust predictors for better survival in colorectal cancer patients treated with cetuximab and metastatic breast cancer patients treated with trastuzumab respectively [40,41]. In recent studies, FcγR polymorphisms in breast cancer patients treated with trastuzumab or neuroblastoma patients treated with anti-GD2 mAbs were directly linked to ADCC amplitude by in vitro studies using patient derived immune cells [42,43]. These multiple analyses confirm that patients with high affinity FcγR that mediate stronger ADCC have better clinical outcomes when administered mAb therapy, irrespective of cancer type or target antigen. In addition to examining FcγR polymorphisms, studies have used patient samples from clinical trials to investigate the relative importance of ADCC to therapeutic success. In one study, patients with HER2-positive breast cancer that were treated with trastuzumab had IHC staining for granzyme B performed on their tumor samples as a surrogate marker of ADCC activity. Patients who received trastuzumab were found to have better overall survival and higher levels of ADCC compared to the other cohorts [44]. Furthermore, patient-derived in vitro models demonstrated ADCC as a major therapeutic mechanism of rituximab in non-Hodgkin lymphoma and anti-CD38 antibodies in multiple myeloma [45,46]. Taken together, there is strong evidence that ADCC plays a critical role in facilitating mAb-based anti-tumor therapeutic responses in patients. In fact, additional variables that would affect ADCC activity such as target antigen expression level and density, mAb isotype, and mAb dose all correlate with clinical response [47]. The ability of mAbs to mediate ADCC is recognized as a major determining factor for mAb therapy success, and research and development of novel mAbs has shifted towards designing mAbs with improved capacity to mediate ADCC. ADCC functionality of antibodies can be enhanced by altering the Fc portion of the mAb to increase their binding affinity to the activating FcγRIIIA via site-directed mutagenesis, changing Fc domain glycosylation, and/or removing Fc domain fucosylation [48,49,50,51]. Next generation mAbs that are afucosylated have shown promise in clinical trials [52].

## 4. Clinical Uses

Over the past thirty years, various forms of mAb-derived treatments have been used clinically in an effort to capitalize on the potential of targeted therapy. Antibodies are extremely versatile as platforms for the development of novel therapeutics which has resulted in a large diversity of approaches. The discovery of targetable tumor-specific antigens fueled interest in designing immunotherapies [53]. Upon the emergence of mAbs, it was thought that using mAbs to target tumor cell antigens might be a more effective and less toxic treatment than traditional chemotherapy. In 1988, scientists identified a protein, CD20, which was specific to mature B cells. CD20 was found to be abundantly expressed on cancerous B cells in non-Hodgkin’s lymphoma, but not found on healthy immature B cells. Therefore, a mAb treatment that targeted CD20 could eliminate the cancerous cells, but immature B cells would remain to replenish the supply of healthy cells. Thus, CD20 became the first target for mAb therapy and the anti-CD20 mAb rituximab was the first mAb to be approved for the treatment of cancer [54]. By targeting antigens identified as overexpressed on solid tumor cells, more mAbs have since demonstrated efficacy as cancer therapeutics. Today, mAbs directed to such targets as epidermal growth EGFR and HER2 see wide use in the clinic for the treatment of colorectal and breast cancers respectively [55,56]. In addition, there is currently a multitude of other ways that mAbs are employed in cancer therapy including antibody-drug conjugates, targeting pro-tumorigenic compounds in the microenvironment, bispecific T cell engagers (BiTEs), and immune checkpoint inhibitors. A comprehensive list of FDA approved mAb-based cancer therapies is provided in Table 1. 

Early efforts in the development of mAb therapies focused on enhancing the direct cytotoxic effects on targeted tumor cells. With the exception of the aforementioned mAbs directed against CD20, HER2, and EGFR, most mAbs have little anti-tumor activity on their own. In spite of this, the specificity of mAbs for tumor antigen makes them useful for delivering cytotoxic compounds directly to tumor cells. Clinically beneficial anti-tumor activity has been accomplished by conjugating mAbs with different effector molecules that cause tumor cell death after antibody binding and internalization. Effector molecules may include cytotoxic drugs, immunotoxins, and radionuclide agents. The most important consideration in antibody–conjugate design is the selection of the target, which is the main determinant of anti-tumor activity and selectivity [58]. In addition, targets must be capable of internalization upon antibody binding in order to release the drug. Brentuximab vedotin became the first antibody–drug conjugate (ADC) to be FDA approved in 2011 [59]. Brentuximab vedotin is a mAb targeting CD30 expressed by lymphoma cells linked to the microtubule destabilizing agent monomethyl auristatin E. Ado-trastuzumab emtansine is an ADC composed of trastuzumab and the cytotoxic maytansine derivative DM1 that was approved in 2013 for patients with metastatic breast cancer that is HER2-positive [60]. Eight ADCs have been approved for use in the treatment of various cancers (Table 1). ADCs targeting mesothelin, DLL3, and GPNMB, among others remain in advanced stages of clinical testing [61]. Research into ADCs continues because of improved understanding of the mechanistic basis of ADC activity that enables rational design of combinations with other cytotoxic payloads and findings indicate that ADCs may also stimulate additional anti-tumor immunity by T cells [62]. The second class of agents that can be delivered to tumor cells via conjugation to mAbs are biologic toxins. This method has proven difficult due to the extreme potency of the toxins, causing unacceptable toxicity in patients. Pseudomonas exotoxin A (PE) and ricin toxins are the most common toxins in targeted cancer therapy and remain under clinical investigation [63]. To date, only moxetumomab pasudotox, a CD22 targeted mAb linked to PE for hairy-cell leukemia, has received FDA approval [64]. The last category of antibody-based compound delivery involves radionuclides. Radioimmunotherapy uses a mAb labeled with a radionuclide as a form of targeted radiation therapy. Currently, only two radioimmunotherapies have been FDA approved: yttrium-90 (90Y)-ibritumomab tiuxetan and iodine-131 (131I)–tositumomab. Both agents utilize a mAb specific for CD20 to deliver either yttrium-90 or iodine-131 to lymphoma cells. Unfortunately, radioimmunotherapies can cause life-threatening systemic toxicity and solid tumors are often inaccessible or insensitive. Since the practicalities of preparing and delivering these agents have proved complex they have not seen widespread use, and tositumomab was discontinued by their parent company [65]. 

The tumor microenvironment contains many factors that are known to inhibit anti-tumor immune responses, promote tumor cell growth, and induce pro-tumorigenic angiogenesis. Targeting these critical pro-tumorigenic processes within the tumor microenvironment has proved clinically efficacious. Historically, the most relevant target has been vascular endothelial growth factor (VEGF), which is abundant in the microenvironment of many solid tumors and binds to its receptor (VEGFR) found on the tumor adjacent vascular endothelium to stimulate angiogenesis. The mAb bevacizumab, which is targets VEGF and blocks VEGF from binding to its receptor, is approved for the treatment of many different cancers [66]. Similar efforts to target VEGFRs using ramucirumab, a mAb to VEGFR2, and icrucumab, a mAb to VEGFR1, have shown promise [67,68]. Other pathways and factors that are proangiogenic such as platelet-derived growth factor (PDGF)/PDGF-receptor (PDGFR) signaling are important therapeutic targets [69]. In addition to proangiogenic targets, transforming growth factor-beta (TGF-β), which is secreted by some tumor cells, inhibits immune effector cell function in the tumor microenvironment [70]. Fresolimumab is a mAb that targets TGF-β and is in ongoing clinical trials [71]. Targeting the tumor microenvironment with mAbs represents a compelling strategy to synergistically inhibit pro-tumorigenic processes when combined with tumor targeted therapy.

**Figure 2:**
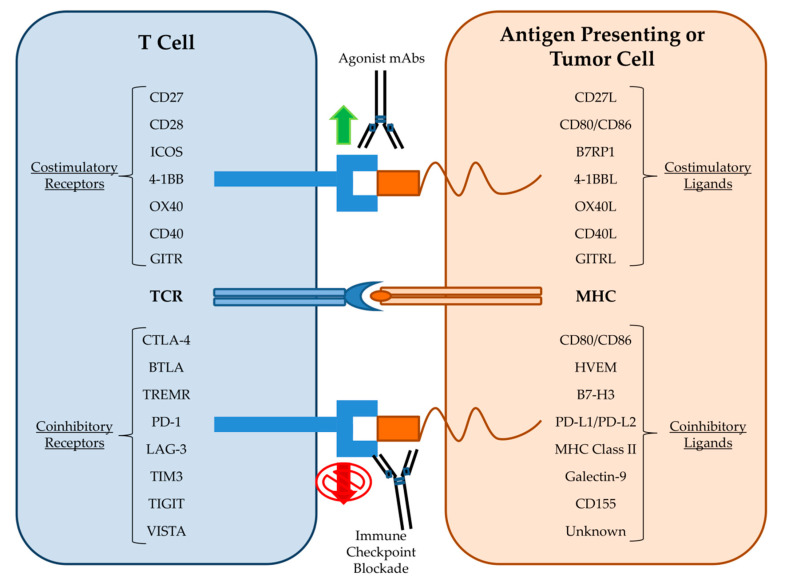
Immune checkpoint targets of monoclonal antibodies.

Recently, the most successful mAb-based strategies have moved away from targeting tumor antigens and instead focused on targeting immune cells in order to enhance their anti-tumor capabilities. One of the first mAb approaches to stimulate T cell anti-tumor immunity was the development of bispecific T Cell Engager (BiTE) antibodies that both target a tumor antigen such as CD19 and the activating receptor, CD3, on T cells. BiTEs combine direct targeting of tumor cells with recruitment of cytotoxic T cells into the tumor microenvironment and led to tumor regressions even when administered at doses three orders of magnitude less than the parent mAb alone [72]. The CD19-CD3 BiTE blinatumomab conferred significant clinical benefit to acute lymphoblastic leukemia patients and was FDA approved in 2017 [73]. Clinical trials are currently underway using BiTEs generated from the widely used anti-HER2 and anti-EGFR mAbs trastuzumab and cetuximab. Other mAb approaches seek to enhance T cell specific immunity against tumor cells by stimulating activating receptors such as 4-1BB, OX40, CD27, CD40, and ICOS (Figure 2). Agonist antibodies towards CD40 stimulate antigen presentation by dendritic cells and mAbs to OX40 and 4-1BB activate T cells while simultaneously dampening the activity of inhibitory T regulatory cells (Tregs) [74]. mAbs designed to stimulate these activating receptors are in various stages of clinical trials both alone and in combination with other immunotherapy approaches. Additional mAbs that deplete inhibitory Tregs directly, such as daclizumab, which targets CD25 on Tregs, are also undergoing clinical trials [75]. 

The most well-known and promising type of mAb therapy for cancer is the blockade of immune checkpoints (Figure 2). Immune cell activation and regulation is a highly complex process that must integrate a variety of costimulatory and coinhibitory signals in order to control immune cell responses to antigen. Immune checkpoints are inhibitory receptors and pathways that are responsible for maintaining self-tolerance and modulating immune responses in order to curtail collateral tissue damage [76]. Cytotoxic T lymphocyte antigen-4 (CTLA-4) was the first T cell checkpoint to be identified. CTLA-4 is mainly expressed by Treg cells but also becomes upregulated on activated T cells where it then outcompetes for binding of the costimulatory ligands CD80 and CD86 [77]. Therefore, it was theorized that blockade of CTLA-4 could indirectly and directly amplify the anti-tumor T cell response by removing inhibitory Tregs and maintaining the activating signals to cytotoxic T cells respectively. Immune checkpoint blockade (ICB) therapy utilizing mAbs against CTLA-4 was then introduced and following success in animal models was rapidly developed for evaluation in clinical trials [78]. In 2011, the FDA approved the first ICB therapy, the anti-CTLA-4 mAb Ipilimumab, based on promising results from a clinical trial in melanoma patients [79]. Ipilimumab remains the subject of clinical trials for use in additional cancer types [80]. Programmed death receptor-1 (PD-1) is another inhibitory immune checkpoint and is associated with the programmed death pathway in T cells [81]. PD-1 is expressed on activated CD8+ T cells, Tregs, and activated B cell and natural killer (NK) cells. PD-1 is considered a major regulator of effector T cell function and is therefore regarded as a key checkpoint target [82]. Tumor cells are known to upregulate the PD-1 ligand, PD-L1, in order to exhaust tumor infiltrating lymphocytes (TILs) [83]. In 2014, the anti-PD-1 mAb nivolumab gained FDA approval for melanoma patients following reports of improved patient outcomes in the CheckMate-037 clinical trial [84]. Subsequent clinical trial successes led to the approval of nivolumab and another anti-PD-1 mAb, pembrolizumab, for the treatment of a wide variety of malignancies [85,86,87]. Additional mAbs targeting either PD-1 (pidilizumab) or its ligands (durvalumab and atezolizumab) have also performed well in clinical trials [88]. As of this writing, mAbs against the immune checkpoints CTLA-4, PD-1, and PD-L1 have received numerous FDA approvals and are used as first-line therapies for the treatment of certain solid tumors [89]. The robust effectiveness of ICB has led to the rapid study of other immune cell inhibitory receptors. Members of the immunoglobulin superfamily such as lymphocyte activation gene 3 (LAG3), T cell immunoglobulin and mucin domain-containing 3 (TIM3), T cell immunoglobulin and immunoreceptor tyrosine-based inhibitory motif domain (TIGIT), and V-domain Ig suppressor of T cell activation (VISTA) are all being explored as potential checkpoint therapeutic targets [90,91]. Importantly, checkpoint blockade also affects other components of the innate immune system such as NK cells. NK cells have the intrinsic ability to kill tumor cells; however, NK cell effector function is modulated by various molecular checkpoints [92]. The blockade of NK cell associated inhibitory receptors such as KIRs are therefore under preclinical investigation [93]. The demonstrable anti-tumor activity and favorable toxicity profile of ICB has cemented mAbs as one of the backbones of cancer therapy.

## 5. Mechanisms of Resistance

Although mAb therapy has proven successful in the treatment of cancer, clinical resistance to these agents continues to be a major issue. Only a minority of patients will respond, with the vast majority developing refractory disease within one year [94,95,96]. Therapeutic resistance can be considered either innate (primary) or acquired (secondary) with differing mechanisms in each scenario. Innate resistance is mainly due to mutations already present in the tumor cells prior to therapy whereas acquired resistance is the result of immune selection pressure and immunoediting of the tumor during therapy. Preclinical models and clinical trials of mAb therapy have unraveled a myriad of mechanisms of resistance; and they include: Mutations of the antibody target, induction of alternative growth signaling pathways, epithelial to mesenchymal transition (EMT), and impaired effector cell responses. 

A limitation of mAb therapy is that efficacy is dependent on tumor cell expression of the target molecules that are able to be bound by the antibodies. While *CD20* gene mutations can confer irreversible resistance to rituximab in lymphoma patients, such mutations were rarely detected at both the initiation of treatment and in cases who relapsed following therapy [97]. A S492R mutation in the EGFR ectodomain imparts resistance to cetuximab but not panitumumab due to their recognition of distinct epitopes [98]. Interestingly, cancer cells expressing EGFR variant III are less sensitive to cetuximab even though the cetuximab binding epitope remains intact [99]. Cell lines chronically exposed to rituximab acquire resistance that is associated with the downregulation of CD20 at both the transcriptional and protein level [100]. Likewise, multiple myeloma patients who received the anti-CD38 monoclonal antibody daratumumab lost CD38 expression in their tumors which correlated with impaired response [101]. Cetuximab-mediated ADCC highly correlates with EGFR surface expression in cell lines but clinical response in patients appear to be independent of tumor EGFR expression level [102,103]. Instead, it has been suggested that mutations and polymorphisms of EGFR are responsible for cetuximab refractory disease. In HNSCC patients that expressed the EGFR-K521 variant (~40% of cases) there was reduced affinity of cetuximab to EGFR and efficacy could only be restored with optimization of ADCC [104]. Similarly, KRAS mutation status may affect susceptibility of EGFR overexpressing cancers to ADCC. Cell lines with mutant KRAS had impaired Fas–Fas ligand interactions that are necessary for induction of target cell apoptosis during ADCC [105]. Downregulation of HER2 expression has been proposed as a mechanism of resistance to trastuzumab-mediated ADCC but it remains a controversial issue [106]. Despite conflicting results from in vitro studies there was no reduction found in HER2 expression in breast cancer patients who received trastuzumab [107]. However, it is known that interferon gamma (IFNγ) exposure can lead to HER2 downregulation through STAT1 mediated pathways [108]. Furthermore, trastuzumab-mediated ADCC induces IFNγ release from NK cells which leads to a STAT1 dependent downregulation of HER2 expression and concomitant resistance to trastuzumab [109]. It is also known that IFNγ-induced activation of STAT1 signaling leads to PD-L1 upregulation on the tumor cell surface that confers resistance to NK cell-mediated ADCC [110].

Mutations of the antibody target and associated downstream signaling molecules can lead to acquired resistance to mAb therapy by activating alternative growth or survival signaling pathways. In colorectal cancers, the most frequent mechanism of cetuximab resistance has been reported as genomic alterations in downstream effectors of EGFR such as *KRAS*, *NRAS*, *BRAF*, and *PIK3CA* [111]. Alterations in these pathways bypass EGFR signaling inhibition by cetuximab. For example, *KRAS* point mutations are causally linked with acquired resistance to cetuximab treatment in colorectal cancer [112]. In metastatic colorectal cancer patients, activating mutations of the oncogenes *RAS*, *BRAF*, and/or *PIK3CA* were identified as significant predictors of primary resistance to cetuximab [113]. The resulting enhanced signaling through the downstream MAPK and PI3K/AKT pathways and increased expression of anti-apoptotic BCL-2 proteins is a main mechanism of resistance to mAb induced apoptosis. Furthermore, NRAS mutations that maintain MAPK signaling prevent cetuximab efficacy by preserving the dysregulated ligandless signaling of the pro-tumorigenic EphA2 receptor [114]. Activation of alternative proliferative and survival pathways such as MAPK and eIF5A2 has also been discovered in HNSCC and hepatocellular carcinoma respectively in response to cetuximab [115,116]. HER2 mutations in breast cancer can confer resistance to trastuzumab; however, trastuzumab is still able to bind the mutant HER2 [117]. Mutant HER2 leads to dysregulation of the PI3K-AKT signaling pathway and enables trastuzumab resistance through similar anti-apoptotic effector molecules. Furthermore, activating mutations of the PI3K/AKT/mTOR pathway also contribute to trastuzumab resistance in breast cancer [118]. Several studies have reported overexpression of compensatory growth factors such as insulin-like growth factor-I receptor or EGFR as additional potential mechanisms of resistance to trastuzumab [119]. Other signaling pathways implicated in trastuzumab resistance include aberrant activation of the tyrosine kinase SRC, cyclin E/cyclin-dependent kinase (CDK) 2, and cyclin D1/CDK4/6 [120]. In one study of esophageal squamous cell carcinoma, trastuzumab resistant tumor clones had a reduced susceptibility to the perforin-granzyme system [121]. Similarly, X-linked inhibitor of apoptosis protein, which is overexpressed in breast cancer, drove resistance to ADCC mediated by both cetuximab and trastuzumab [122]. In an in vitro study of rituximab-resistant lymphoma clones, major survival pathways such as NF-κB and ERK1/2 became constitutively hyper-activated after treatment, which led to overexpression of factors such as Bcl-2, Bcl-xL, and Mcl-1 that prevented the induction of apoptosis by rituximab [123]. 

Epithelial to mesenchymal transition (EMT) is a process in which cancer cells lose their epithelial phenotype characterized by cell-to-cell adhesion and instead gain the invasive properties of mesenchymal cells. In preclinical models, EMT was uncovered as a possible mechanism of cetuximab resistance [124]. The induction of EMT was later confirmed to occur early on in head and neck cancer patients receiving cetuximab. Multiple subsequent studies have revealed EMT mediates acquired resistance to cetuximab via a myriad of mechanisms which include loss of EGFR expression [125,126,127]. Moreover, activation of the EMT pathway is a key predictor of cetuximab resistance in colorectal cancer [128]. In breast cancer, molecular features associated with EMT are linked to primary resistance to trastuzumab [129]. Additionally, sustained treatment of HER2 positive/PTEN negative breast cancers with trastuzumab induced EMT in a subset of patients which conferred acquired resistance [130].

ADCC is considered a main therapeutic mechanism of mAb therapy, and clinical resistance often involves impaired cytotoxic immune effector cell responses. Capuano et al. described a novel mechanism of immune exhaustion, whereby NK cells chronically exposed to rituximab lost their cytotoxic functions due to CD16 ligation [131]. NK cell checkpoints can also regulate ADCC. Poliovirus receptor-like receptors such as TIGIT are known to be involved in trastuzumab-mediated ADCC of cancer cells by NK cells, and blockade of those receptors was able to enhance trastuzumab based responses in breast cancer patients [132]. Finally, NK cell-mediated ADCC is also dependent on the expression of several proteins that are important members of the immune synapse. ADCC is partly dependent on recognition through ICAM-1 and CD18, though this appears to be less important for trastuzumab-mediated ADCC [133]. Cancer cell loss of ligands for the activating NKG2D receptor on NK cells such as MICA and dysregulation of the NKG2A-HLA-E axis can also prevent NK cell initiation of ADCC [134]. A recently reported novel mechanism of resistance to ADCC involves the downregulation of multiple cell surface proteins associated with the immune synapse in response to cetuximab and trastuzumab [135]. 

## 6. Combination Therapies

While mAb has success as a monotherapy in some patients, treatment paradigms are trending towards employing them as combinations with chemotherapy, radiation, molecularly targeted drugs such as tyrosine kinase inhibitors, other antibodies against the same target, immune checkpoint inhibitors, vaccines, and/or cellular therapies. These many combination strategies are currently undergoing both preclinical investigation and clinical trials and this vast field is more exhaustively covered elsewhere [136]. In this section, we will briefly cover combination therapies involving multiple monoclonal antibodies. It is now widely recognized that the mechanism of action of monoclonal antibodies includes an immune effector cell component. In particular, cetuximab efficacy has been partly attributed to ADCC, which can link innate and adaptive anti-tumor immune responses. Destruction of tumor cells via NK cell-mediated ADCC releases tumor cell specific proteins that when presented by antigen presenting cells to cytotoxic T cells leads to a more effective anti-tumor response. Head and neck squamous cell carcinoma (HNSCC) patients with durable responses to cetuximab have sustained anti-tumor specific immune responses [137]. With the rise of immune checkpoint inhibitors that can further potentiate such immune responses, it is hypothesized that ICB may act in a synergistic manner with cetuximab. There is growing to support combining anti-PD-1/PD-L1 mAbs with cetuximab in HNSCC patients [138]. Additionally, combinations of either pembrolizumab or avelumab with cetuximab are currently in clinical trials [NCT03082534, NCT03082534]. Likewise, the use of ICB in breast cancer in order to enhance anti-HER2 mAb therapies is a promising strategy. In fact, preclinical evidence suggests that resistance to trastuzumab monotherapy can be overcome by combination with ICB [139]. Based on those results several clinical trials were formed to investigate the relationship between ICB and HER2-targeted mAbs [140]. Preliminary results from the phase I/II PANACEA trial, which tested pembrolizumab combined with trastuzumab in treating breast cancer patients who overexpressed HER2, indicated synergy in the PD-L1+ patient subset [141].

Although there are many immune checkpoints of T-cell activation, each checkpoint has distinct mechanisms. Consequently, ICB combinations that target multiple checkpoints will enhance T cell responses in a synergistic manner. The combination of mAbs targeting CTLA-4 and PD-1 performed significantly better in preclinical mouse models than either antibody alone [142]. Similarly, in metastatic melanoma patients combined therapy of ipilimumab and nivolumab was found to be more effective than either treatment used as a monotherapy [143]. The FDA has since approved the combination of ipilimumab and nivolumab for melanoma. As the first ICB combination with FDA approval, ongoing clinical trials continue to evaluate ipilimumab plus nivolumab in other cancer types.

Anti-PD-1 mAbs are most often used in combinatorial strategies due to their more favorable toxicity profile in contrast to anti-CTLA-4 monoclonal antibodies. The immune checkpoints LAG3 and TIM3 are commonly found co-expressed with PD-1 on exhausted T cells. ICB of LAG3 combined with anti-PD-1 is undergoing clinical trial in glioblastoma (NCT02658981) and other cancers (NCT02460224). There are similar clinical trials for the combination of anti-TIM3 and anti-PD-1 antibodies in liver cancer (NCT03680508) and several other solid tumors (NCT03744468). Another promising combination strategy involves uniting ICB with agonistic antibodies that activate stimulatory receptors. 4-1BB is a costimulatory receptor found on T cells and NK cells and clinical trials that evaluate 4-1BB agonist antibodies in combination with anti-PD-1 mAb therapy are underway (NCT02253992 and NCT02179918). An agonist antibody to the glucocorticoid-induced tumor necrosis factor receptor–related protein (GITR), which promotes T cell activation, also proved to be successful when combined with nivolumab [144]. Additional mAb combinations that include agonist antibodies to OX40, which only becomes expressed on activated T cells, are the subject of multiple clinical trials (NCT01714739 and NCT01750580).

## 7. Concluding Remarks

Monoclonal antibody therapy has only recently become one of the major modalities for the treatment of cancer. Many of the mechanisms of action and their clinical relevance remain poorly understood. Despite the notable clinical successes of antibody therapy, therapeutic resistance remains a major challenge. Future studies must focus on analysis of the mechanisms of action of mAbs in order to identify new approaches to increase clinical efficacy. For instance, studies have revealed that ADCC plays a major role in mediating mAb responses and therefore engineering strategies that augment ADCC activity represents a promising future approach. Combinations of tumor targeted mAbs with ICB have demonstrated that there are several encouraging avenues for maximizing the clinical benefit of mAb therapy. Additionally, mutations of both the antibody target and any associated signaling pathways are important biomarkers of mAb efficacy and resistance. Future mAb treatment strategies must incorporate inhibitors of these alternative signaling pathways in order to abrogate resistance. Treatment paradigms involving monoclonal antibodies will continue to evolve and have the potential to offer curative therapy for many cancer patients. 

## Figures and Tables

**Table 1:** FDA-approved monoclonal antibodies for cancer.

Name	Antigen	Format	Indications (Year of First Approval) ^1^
**Unconjugated Antibodies**			
Atezolizumab	PD-L1	Humanized IgG1	Bladder, Non-small cell lung (2016), and Triple-negative breast (2019) cancers (2019)
Avelumab	PD-L1	Human IgG1	Urothelial Carcinoma (2017) and Merkel Cell Carcinoma (2017)
Bevacizumab	VEGF	Humanized IgG1	Colorectal (2004), Non-small cell lung (2006), Renal (2009), Glioblastoma (2009), and Ovarian (2018) Cancers
Cemiplimab	PD-1	Human IgG4	Cutaneous squamous-cell carcinoma (2018)
Cetuximab	EGFR	Chimeric IgG1	Colorectal cancer (2004) and Head and neck squamous cell carcinoma (2006)
Daratumumab	CD38	Human IgG1	Multiple Myeloma (2015)
Dinutuximab	GD2	Chimeric IgG1	Neuroblastoma (2015)
Durvalumab	PD-L1	Human IgG1	Bladder Cancer (2017)
Elotuzumab	SLAMF7	Humanized IgG1	Multiple Myeloma (2015)
Ipilimumab	CTLA-4	Human IgG1	Melanoma (2011) and Renal cell carcinoma (2018)
Isatuximab	CD38	Chimeric IgG1	Multiple Myeloma (2020)
Mogamulizumab	CCR4	Humanized IgG1	Cutaneous T-cell lymphoma (2018)
Necitumumab	EGFR	Human IgG1	Non-small cell lung cancer (2015)
Nivolumab	PD-1	Human IgG4	Melanoma (2014), Lung (2015), and Renal (2018) cancers
Obinutuzumab	CD20	Humanized IgG2	Chronic lymphocytic leukemia (2013)
Ofatumumab	CD20	Human IgG1	Chronic lymphocytic leukemia (2014)
Olaratumab	PDGFRα	Human IgG1	Sarcoma (2016)
Panitumumab	EGFR	Human IgG2	Colorectal Cancer (2006)
Pembrolizumab	PD-1	Humanized IgG4	Melanoma (2014), Various (2015-)
Pertuzumab	HER2	Humanized IgG1	Breast cancer (2012)
Ramucirumab	VEGFR2	Human IgG1	Gastric cancer (2014)
Rituximab	CD20	Chimeric IgG1	B-Cell Lymphoma (1997)
Trastuzumab	HER2	Humanized IgG1	Breast cancer (1998)
**Antibody–Drug Conjugates (ADCs)**			
Gemtuzumab ozogamicin	CD33	Humanized ADC	Acute myeloid leukemia (2000)
Brentuximab vedotin	CD30	Chimeric ADC	Hodgkin’s lymphoma and Anaplastic large-cell lymphoma (2011)
Trastuzumab emtansine	HER2	Humanized ADC	Breast cancer (2013)
Inotuzumab ozogamicin	CD22	Humanized ADC	Acute lymphoblastic leukemia (2017)
Polatuzumab vedotin	CD79B	Humanized ADC	B-Cell Lymphoma (2019)
Enfortumab vedotin	Nectin-4	Human ADC	Bladder cancer (2019)
Trastuzumab deruxtecan	HER2	Humanized ADC	Breast cancer (2019)
Sacituzumab govitecan	TROP2	Humanized ADC	Triple negative breast cancer (2020)
Moxetumomab pasudotox	CD22	Mouse ADC	Hairy-cell leukemia (2018)
Ibritumomab tiuxetan	CD20	Mouse IgG1-Y90 or In111	Non-Hodgkin’s lymphoma (2002)
Iodine (I131) tositumomab	CD20	Mouse IgG2-I131	Non-Hodgkin’s lymphoma (2003)
Blinatumomab	CD19, CD3	Mouse BiTE	Acute lymphoblastic leukemia (2014)

^1^ Indications and year of first approval for each antibody were accessed using the FDA drug database [57].
